# Combination of epigenetic erasing and mechanical cues to generate human epiBlastoids from adult dermal fibroblasts

**DOI:** 10.1007/s10815-023-02773-4

**Published:** 2023-03-18

**Authors:** Georgia Pennarossa, Sharon Arcuri, Teresina De Iorio, Sergio Ledda, Fulvio Gandolfi, Tiziana A. L. Brevini

**Affiliations:** 1 Department of Veterinary Medicine and Animal Science, Center for Stem Cell Research, Laboratory of Biomedical Embryology and Tissue Engineering, Università Degli Studi Di Milano, 26900 Lodi, Italy; 2 Department of Veterinary Medicine, University of Sassari, 07100 Sassari, Italy; 3 Department of Agricultural and Environmental Sciences - Production, Landscape, Agroenergy, Università degli Studi di Milano, 20133 Milan, Italy

**Keywords:** 3D culture systems, epiBlastoids, Epigenetic erasing, ICM-like cells, Mechanosensing-related cues, TR-like cells

## Abstract

**Purpose:**

This study is to develop a new protocol that combines the use of epigenetic cues and mechanical stimuli to assemble 3D spherical structures, arbitrarily defined “epiBlastoids,” whose phenotype is remarkably similar to natural embryos.

**Methods:**

A 3-step approach is used to generate epiBlastoids. In the first step, adult dermal fibroblasts are converted into trophoblast (TR)-like cells, combining the use of 5-azacytidine, to erase the original phenotype, with an ad hoc induction protocol, to drive cells towards TR lineage. In the second step, epigenetic erasing is applied once again, in combination with mechanosensing-related cues, to generate inner cell mass (ICM)-like organoids. Specifically, erased cells are encapsulated into micro-bioreactors to promote 3D cell rearrangement and boost pluripotency. In the third step, TR-like cells are co-cultured with ICM-like spheroids in the same micro-bioreactors. Subsequently, the newly generated embryoids are transferred to microwells to favor epiBlastoid formation.

**Results:**

Adult dermal fibroblasts are successfully readdressed towards TR lineage. Cells subjected to epigenetic erasing and encapsulated into micro-bioreactors rearrange in 3D ICM-like structures. Co-culture of TR-like cells and ICM-like spheroids into micro-bioreactors and microwells induces the formation of single structures with uniform shape reminiscent in vivo embryos. CDX2^+^ cells localized in the out layer of the spheroids, while OCT4^+^ cells in the inner of the structures. TROP2^+^ cells display YAP nuclear accumulation and actively transcribed for mature TR markers, while TROP2^−^ cells showed YAP cytoplasmic compartmentalization and expressed pluripotency-related genes.

**Conclusion:**

We describe the generation of epiBlastoids that may find useful application in the assisted reproduction field.

## Introduction

Early mammalian embryogenesis encompasses several events that lead to the formation of the blastocyst that comprises two distinct structures: the trophectoderm (TE) and inner cell mass (ICM) [1]. The TE consists of trophoblast (TR) cells that contribute to the formation of the placenta, nourishing and supporting the fetus during intrauterine life [2–4]. The ICM contains pluripotent cells that are able to differentiate in the three definitive germ layers, giving rise to all tissues of the embryo proper [5].

The process of development during which embryonic cells specialize and differentiate are driven by molecular mechanisms and mechanical cues that have long aroused great interest. However, due to the in vivo microenvironment inaccessibility, the material paucity, and the ethical and legal issues, the study of the peri-implantation period in human remains a daunting task [6].

During the last few years, several efforts have been directed towards the creation of in vitro models that recapitulate embryogenesis in vivo. In this context, the successful and scalable generation of blastocyst-like structures (termed blastoids), using different methods and cell sources, have been reported in both mouse and human [7–13].

**Fig. 1:**
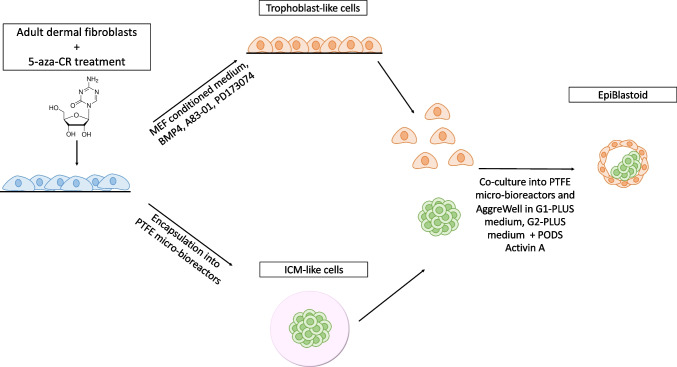
Scheme illustrating epiBlastoid generation from adult dermal fibroblasts

Here, we describe a new 3-step approach that allows the generation of 3D multicellular spherical structures, remarkably similar to natural embryos (Fig. 1). To this purpose, starting from easily accessible terminally differentiated cells, we combine the use of epigenetic cues and mechanical stimuli. In particular, in the first step, we convert adult dermal fibroblasts into TR-like cells, using 5-azacytidine (5-aza-CR) to erase the original cell phenotype, and apply an ad hoc induction protocol to drive cells into the TR lineage [14, 15]. In the second step, we combine epigenetic and mechanosensing-related stimuli to generate ICM-like spheroids. Specifically, we apply the same epigenetic erasing protocol and encapsulate cells into polytetrafluoroethylene (PTFE) micro-bioreactors to promote 3D cell rearrangement and boost pluripotency [16, 17]. In the third and last step, we co-culture chemically induced TR-like cells with ICM-like spheroids in the same micro-bioreactor and, subsequently, into microwells, to further encourage differentiation and favor the formation of 3D spherical structures resemble in vivo blastocysts and that we arbitrarily defined “epiBlastoids.”

The results obtained demonstrate that the approach here described allows for the generation of a blastocyst-like model that is able to mimic the physiological organization of early embryos in vivo and may find useful application in the assisted reproduction field.

## Materials and methods

All reagents were purchased from Thermo Fisher Scientific unless otherwise indicated.

### Ethical statement

Human cell isolation from healthy adult individuals was approved by the Ethical Committee of the Ospedale Maggiore Policlinico, Milano (CE 479_20071.6). All experiments were performed in accordance with the approved guidelines.

### Isolation and culture of human dermal fibroblasts

Human fibroblasts were isolated from fresh skin biopsies of 6 healthy individuals (3 women and 3 men). Tissue fragments of approximately 2 mm^3^ were transferred onto petri dishes (Sarstedt) previously coated with 0.1% porcine gelatin (Sigma-Aldrich) and cultured in Dulbecco’s modified Eagle’s medium (DMEM) supplemented with 20% Fetal Bovine Serum (FBS), 2 mM glutamine (Sigma-Aldrich), and antibiotics (Sigma-Aldrich). After 6 days of culture, fibroblasts grown out of the original skin explants and fragments were carefully removed. Cells were subsequently cultured in fibroblast culture medium (FCM) composed of DMEM, 10% FBS, 2 mM glutamine (Sigma-Aldrich), and antibiotics (Sigma-Aldrich) and maintained in 5% CO_2_ at 37 °C. Passages were carried out twice a week at 1:3 ratio. From each individual (*n* = 6) were obtained one primary cell line that was used at least in triplicate in 3 independent experiments.

### Generation of TR-like cells through 5-aza-CR exposure and trophoblast induction

Cells at passages between 6 and 8 were plated into 0.1% gelatin (Sigma-Aldrich) pre-coated 4-well multidishes (Nunc) at concentration of 7.8 × 10^4^ cells/cm^2^. Based on our previous studies [15–24], 24 h after seeding, fibroblasts were treated with 1 µM 5-aza-CR (Sigma-Aldrich) for 18 h. At the end of 5-aza-CR exposure, cells were incubated in Embryonic Stem Cell (ESC) medium consisting of DMEM-low glucose: HAM’S F10 (1:1), 5% FBS, 10% K.O. serum, 2 mM glutamine (Sigma-Aldrich), 0.1 mM β-mercaptoethanol (Sigma-Aldrich), nucleoside mix, 1% non-essential amino acids, 1000 IU/ml ES-growth factor (LIF, Chemicon), and 5 ng/ml b-FGF (R&D System) [19, 25, 26] in 5% CO_2_ at 37 °C for 3 h. TR differentiation was then induced using mouse embryonic fibroblast (MEF) conditioned medium—obtained by culturing 2 × 10^4^ (cells/cm^2^) inactivated MEF in ESC medium without b-FGF for 24 h—supplemented with 10 ng/ml Bone Morphogenetic protein 4 (BMP4, Sigma-Aldrich), 1 µM activin/nodal signaling inhibitor (A83-01, Sigma-Aldrich), and 0.1 µM basic fibroblast growth factor (FGF2) signaling inhibitor (PD173074, Sigma-Aldrich) [17, 27, 28]. Cells were maintained in low O_2_ condition (5% O_2_, 5% CO_2_, and 90% N_2_ atmosphere) at 37 °C for 11 days. Culture medium was refreshed every other day.

### Creation of ICM-like spheroids through 5-aza-CR exposure and cell encapsulation in PTFE micro-bioreactors

Fibroblasts at passages between 6 and 8 were exposed to 1 μM 5-aza-CR (Sigma-Aldrich) and encapsulated in PTFE (Sigma-Aldrich) micro-bioreactors for 18 h [16, 17]. More in detail, a PTFE powder bed with particle size of 1 μm (Sigma-Aldrich 430,935) was created inside a petri dish (Sarstedt), and 1 × 10^4^ cells/30 μl of 1 μM 5-aza-CR in FCM was dispensed on it. The dish was gently rotated in a circular motion, and the powder particles completely covered the surface of the liquid drop, generating the micro-bioreactors. These latters were then transferred in a new petri dish and incubated in 5% CO_2_ at 37 °C_,_ using a humidified chamber to avoid dehydration.

### Production of epiBlastoids by assembling TR-like cells and ICM-like spheroids

TR-like cells differentiated for 11 days were detached from culture supports, centrifuged at 150 g for 5 min, and resuspended in G1-PLUS medium (Vitrolife) to obtain 3 × 10^4^ cells/30 μL. In parallel, ICM-like spheroids were recovered by puncturing with a needle the PTFE micro-bioreactors and transferred in a drop of G1-PLUS medium (Vitrolife). A 40 μL drop containing 3 × 10^4^ TR-like cells (30 μL) and one single ICM-like spheroid (10 μL) was dispensed onto new PTFE powder bed. The newly obtained PTFE micro-bioreactors were maintained in culture for 2 days at 37 °C in 5% CO_2_. Subsequently, the generated epiBlastoids were collected, transferred into non-adherent microwells (AggreWell™, Stemcell technologies) and grown in G2-PLUS medium (Vitrolife). After 24 h of culture in non-adherent microwells, 10^5^ PODS Activin A (Cell guidance systems) were resuspended into 150 μl G2-PLUS medium (Vitrolife). EpiBlastoids were maintained in 5% CO_2_ at 37 °C and kept in culture for additional 4 days.

### Morphometric evaluation

EpiBlastoids were observed under an Eclipse TE200 microscope (Nikon), equipped with a digital camera (Nikon). Pictures were acquired with NIS-Elements Software (Version 4.6; Nikon). Spheroid diameters were measured using ImageJ software (ImageJ software version 1.53j).

### EpiBlastoid cell separation

EpiBlastoids were dissociated to single cell suspension by a double enzymatic digestion with collagenase IV (300 U/ml, Sigma) for 30 min and trypsin–EDTA solution (Sigma) for 20 min, followed by mechanical dissociation by pipetting. Cell suspension was filtered with a 30 µm nylon mesh (Pre-Separation Filters, 30 μm, # 130–041-407, Miltenyi Biotec) and centrifuged at 300 g for 5 min. Supernatants were removed, and trophoblast cell surface antigen 2 (TROP2)^+^ cells were isolated using Magnetic-Activated Cell Sorting (MACS, Miltenyi Biotec) protocol, following the manufacture’s instruction. TROP2^−^ cells were isolated by applying the same protocol and collecting the flow-through. The two cell populations obtained were subjected to gene expression and immunostaining analysis.

### Gene expression analysis

TaqManGene Expression Cells-to-CT kit was used to extract RNA, following the manufacturer’s instruction, and DNase I was added in lysis solution at 1:100 concentration. Quantitative real-time PCR was performed with CFX96 Real-Time PCR detection system (Bio-Rad Laboratories) using the predesigned gene-specific primers and probe sets from TaqManGene Expression Assays listed in Table 1. *GAPDH* and *ACTB* were used as internal reference genes. Target gene quantification was carried out with CFX Manager software (Bio-Rad Laboratories). cDNA obtained from JAR cell line was used as positive controls for TR-like and TROP2^+^ cells. cDNA obtained from the human ESC line HES7 was used as positive controls for ICM-like spheroids and TROP2^−^ cells.

**Table 1:** List of primers used for quantitative PCR

Gene	Description	CAT.N
*ACTB*	Actin, beta	Hs01060665_g1
*CDX2*	Caudal type homeobox 2	Hs01078080_m1
*CGA*	Glycoprotein hormones, alpha polypeptide	Hs00985275_g1
*CGB*	Chorionic gonadotropin beta	Hs03407524_uH
*CYP11A1*	Cytochrome P450 family 11 subfamily A member 1	Hs00167984_m1
*DPPA2*	Developmental pluripotency associated 2	Hs00414521_g1
*ESRRB*	Estrogen related receptor beta	Hs01584024_m1
*GAPDH*	Glyceraldehyde-3-phosphate dehydrogenase	Hs02786624_g1
*GATA2*	GATA binding protein 2	Hs00231119_m1
*GATA3*	GATA binding protein 3	Hs00231122_m1
*GCM1*	Glial cells missing homolog 1	Hs00172692_m1
*HSD17B1*	Hydroxysteroid 17-beta dehydrogenase 1	Hs00166219_g1
*KLF17*	KLF transcription factor 17	Hs00702999_m1
*KRT19*	Keratin 19	Hs00761767_s1
*NANOG*	Nanog homeobox	Hs02387400_g1
*OCT4*	POU Class 5 homeobox 1	Hs04260367_gH
*PGF*	Placental growth factor	Hs00182176_m1
*PRDM14*	PR domain zinc finger protein 14	Hs01119056_m1
*REX1*	ZFP42 zinc finger protein	Hs01938187_s1
*SOX2*	SRY-Box transcription factor 2	Hs04234836_s1
*THY1*	Thy-1 cell surface antigen	Hs06633377_s1
*VIM*	Vimentin	Hs00958111_m1

### Western blot analysis

Constitutive proteins were extracted from cell lysates using the ReadyPrep Protein Extraction Kit (Bio-Rad). Cell nuclear extracts were isolated using the NXtract CelLytic NuCLEAR Extraction Kit (Sigma-Aldrich). Protein concentration was measured by Coomassie Blue-G Dye-binding method. Also, 100 μg of proteins were resuspended in sample buffer (1:1) consisting of 4% SDS (Sigma-Aldrich), 10% 2-mercaptoethanol (Sigma-Aldrich), 20% glycerol (Sigma-Aldrich), 0.004% bromophenol blue (Sigma-Aldrich), and 0.125 M Tris–HCl (Sigma-Aldrich) at pH 6.8. Equal amounts of proteins were loaded, electrophoresed on SDS–polyacrylamide gels, transferred onto 0.45 μm pore size nitrocellulose membranes (Hybond-C Extra, GE Healthcare Life Sciences), and probed with YAP primary antibody (1:1000, Cell signaling, 14,074). Protein bands were visualized by the WesternBreeze chemiluminescent kit, and densitometric analysis was performed using the ImageJ software (ImageJ software version 1.53j). GAPDH (1:1000, Abcam, ab8245) was used as loading control for protein normalization.

### Immunostaining

TR-like cells, ICM-like spheroids, and epiBlastoids were fixed in 4% paraformaldehyde for 20 min, washed three times in PBS, permeabilized with 0.5% Triton X-100 (Sigma) for 30 min, and treated with a blocking solution containing 10% goat serum (Sigma) for 30 min. When cells formed spherical structures, these were dissociated and attached to slides, using a cytocentrifuge (Cytospin 4, Thermo Shandon). Primary antibodies for CDX2 (1:50, Santa Cruz Biotechnology, sc-166830), KRT19 (1:500, Abcam, ab76539), and OCT4 (1:50, Chemicon, ab3209) were incubated overnight at + 4 °C. The day after, samples were washed three times in PBS and incubated with the appropriate secondary antibodies (Alexa Fluor) for 45 min at room temperature using a 1:250 dilution. Nuclei were stained with 4′,6-diamidino-2-phenylindole (DAPI). At the end of the immunostaining procedure, cells were analyzed under an Eclipse E600 microscope (Nikon) equipped with a digital camera (Nikon), and epiBlastoids were transferred and mounted to glass slides and visualized under an Eclipse E600 microscope (Nikon) equipped with a digital camera (Nikon). Pictures were acquired with NIS-Elements Software (Version 4.6; Nikon).

For YAP localization, TROP2^+^ and TROP2^−^ cells (see above) were attached to slides using the cytocentrifuge Cytospin 4 (Thermo Shandon). Cells were then fixed in 4% paraformaldehyde (Sigma), washed three times in PBS, permeabilized, and blocked with a solution containing 0.3% Triton X-100 (Sigma) and 5% serum for 1 h. YAP primary antibody (1:100, Cell signaling, 14,074) was incubated overnight at + 4 °C. Cells were then incubated with suitable secondary antibody (Alexa Fluor) for 45 min. Nuclei were stained with DAPI (Sigma). Samples were observed under a Nikon Eclipse TE200 (Nikon) equipped with a digital camera (Nikon). Pictures were acquired with NIS-Elements Software (Version 4.6; Nikon).

### Cell counting

The number of CDX2, KRT19, and OCT4 immuno-positive cells was counted in 10 randomly selected fields at 200 × total magnification. A minimum of 350 cells were scored in at least three independent replicates. The number of positively stained cells was expressed as a percentage of the total cell counted.

### Statistical analysis

Statistical analysis was performed using the Student *t*-test (SPSS 19.1; IBM). Data were presented as mean ± standard deviation (SD). Differences of *p* ≤ 0.05 were considered significant and were indicated with different superscripts.

## Results

### Generation of TR-like cells through 5-aza-CR exposure and trophoblast induction

**Fig. 2:**
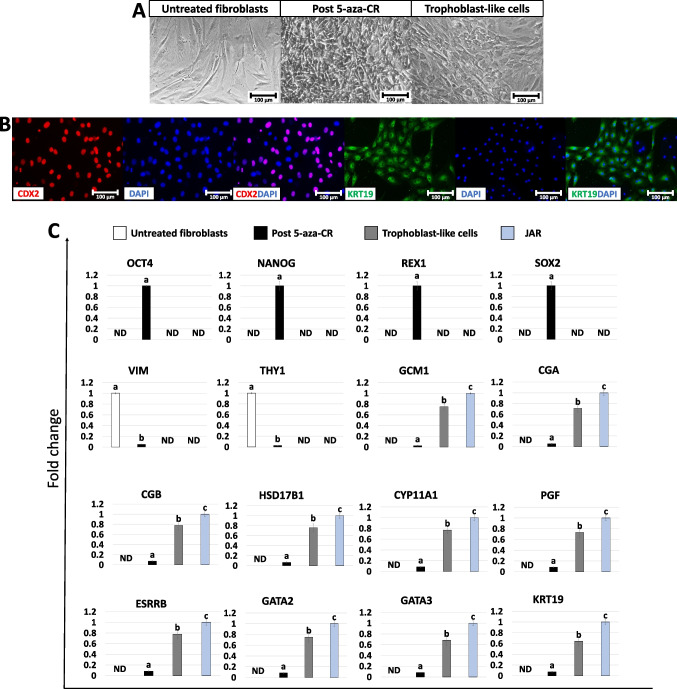
Generation of TR-like cells through 5-aza-CR exposure and trophoblast induction. Fibroblasts exposed to 5-aza-CR lost their typical elongated shape (untreated fibroblasts) and became smaller with larger nuclei and granular cytoplasm (post 5-aza-CR) (scale bars 100 µm; **A**). At day 11 of trophoblast induction, cells acquired a tight adherent epithelial morphology, exhibiting round or ellipsoid shape, with round nuclei and well-defined borders (trophoblast-like cells; scale bar 100 µm; **A**). Immunostainings show cell positivity for the TR markers CDX2 (red, left panel) and KRT19 (green, right panel). Nuclei are stained in blue (scale bars 100 μm; **B**). Transcription levels for pluripotent- (*OCT4*, *NANOG*, *REX1*, *SOX2*, *KLF17*, *PRDM14*, *DPPA2*), fibroblast- (*VIM*, *THY1*), and TR- related genes (*GCM1*, *CGA*, *CGB*, *HSD17B1*, *CYP11A1*, *PGF*, *ESRRB*, *GATA2*, *GATA3*, *KRT19*) in untreated fibroblasts (white bars), fibroblasts exposed to 5-aza-CR (Post 5-aza-CR, black bars), at day 11 of trophoblast induction (trophoblast-like cells, grey bars) and in JAR cell line (JAR, blue bars). Gene expression values are reported with the highest expression set to 1 and all others relative to this. Different superscripts denote significant differences (*P* < 0.05; **C**)

Fibroblasts obtained from skin biopsies grew out of the original explants within 6 days of culture forming a monolayer with cells displaying the standard elongated morphology (Fig. 2A, Untreated fibroblasts). After 5-aza-CR exposure, cell phenotype changed, and treated fibroblasts acquired an oval or round shape, becoming smaller with larger nuclei, granular and vacuolated cytoplasm (Fig. 2A, Post 5-aza-CR). These changes were accompanied by the onset of the pluripotency-related genes *OCT4*, *NANOG*, *REX1*, *SOX2*, *KLF17*, *PRDM14*, and *DPPA2* which were undetectable in untreated fibroblasts (Fig. 2C). In agreement with these observations, epigenetically erased cells significantly downregulated the typical fibroblast markers *VIM* and *THY1* (Fig. 2C). At the end of 11-day TR induction, cells acquired a mature TR morphology, exhibiting round or ellipsoid shape, round nuclei, and well-defined borders (Fig. 2A, trophoblast-like cells). Consistent with these morphological changes, TR-like cells actively transcribed for TR related genes, namely, *GCM1*, *CGA*, *CGB*, *HSD17B1*, *CYP11A1*, *PGF*, *ESRRB*, *GATA2*, *GATA3*, and *KRT19* which were originally absent in untreated fibroblasts (Fig. 2C). This was further support by immunocytochemical results, showing cell positivity for the TR markers CDX2 and KRT19 (79.5 ± 2.99% and 78.65 ± 6.01%, respectively; Fig. 2B).

### Creation of ICM-like spheroids through 5-aza-CR exposure and cell encapsulation in PTFE micro-bioreactors

**Fig. 3:**
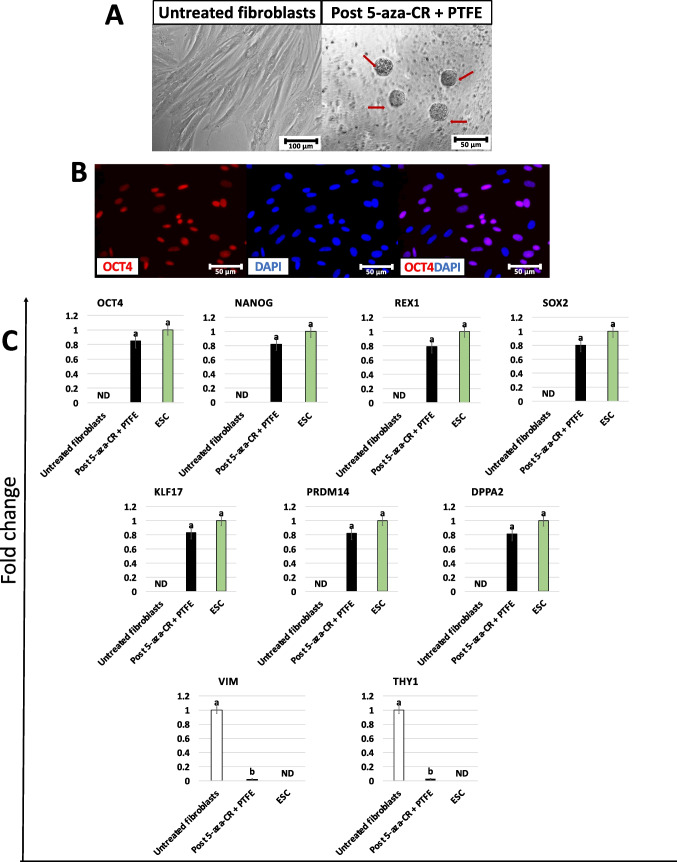
Creation of ICM-like spheroids through 5-aza-CR exposure and cell encapsulation in PTFE micro-bioreactors. Fibroblasts (untreated fibroblasts; scale bar 100 μm) treated with 5-aza-CR and encapsulated in PTFE micro-bioreactors form 3D spherical structures (red arrows, Post 5-aza-CR + PTFE; scale bar 50 μm; **A**). Immunostainings show cell positivity for the pluripotency-related marker OCT4. Nuclei are stained in blue (scale bars 50 μm; **B**). Transcription levels for pluripotent- (*OCT4*, *NANOG*, *REX1*, *SOX2*, *KLF17*, *PRDM14*, *DPPA2*) and fibroblast-related genes (*VIM*, *THY1*) in untreated fibroblasts (white bars), fibroblasts exposed to 5-aza-CR and encapsulated in PTFE micro-bioreactors (post 5-aza-CR + PTFE, black bars), and human ESC line (ESC, green bars). Gene expression values are reported with the highest expression set to 1 and all others relative to this. Different superscripts denote significant differences (*P* < 0.05) (**C**)

After 5-aza-CR exposure and encapsulation in PTFE, fibroblasts became rounded, with large and granulated nuclei, lost their monolayer distribution, and rearranged in 3D spherical structures (Fig. 3A, post 5-aza-CR + PTFE). Consistently with this, the obtained ICM-like spheroids actively transcribed for the main pluripotency-related genes *OCT4*, *NANOG*, *REX1*, *SOX2*, *KLF17*, *PRDM14*, and *DPPA2* originally absent in untreated cells (untreated fibroblasts), while significantly decreased the expression of the fibroblast-specific markers *VIM* and *THY1* (Fig. 3C). In addition, immunocytochemical studies demonstrate the presence of 86.31 ± 4.13% OCT4^+^ cells (Fig. 3B).

### Production of epiBlastoids by assembling TR-like cells and ICM-like spheroids

**Fig. 4:**
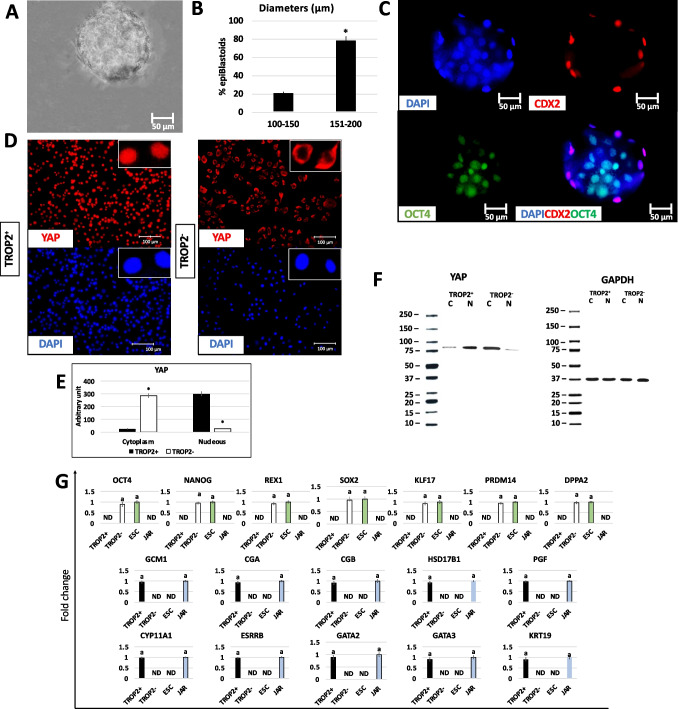
Production of epiBlastoids by assembling TR-like cells and ICM-like spheroids. Representative image of an epiBlastoid (scale bar 50 μm; **A**). Rates of epiBlastoids displaying diameters ranging from 100 to 150 μm and from 151 to 200 μm *Superscripts denote significant differences (*P* < 0.05; **B**). Immunostaining showing CDX2^+^ cells (red) localize in out layer of the epiBlastoids and OCT4^+^ cells (green) in the inner compartment. Nuclei are stained in blue (scale bars 50 μm; **C**). YAP protein is confined in the nuclear compartment of TROP2^+^ cells, while it is excluded from the nucleus and shifts into the cytoplasm in TROP2^−^ cells. Nuclei are stained in blue (scale bars 100 μm; **D**). Densitometric analysis of western blots for YAP protein in cytoplasm (C) and nucleus (N) of TROP2^+^ (black bars) and TROP2^−^ cells (white bars). The values (arbitrary units) are reported as relative optical density of the bands normalized to GAPDH. *Superscripts denote significant differences (*P* < 0.05; **E**). Representative YAP and GAPDH western blots for the two cell compartments of each cell type are also shown (**F**). Transcription levels for pluripotency- (*OCT4*, *NANOG*, *REX1*, *SOX2*, *KLF17*, *PRDM14*, *DPPA2*) and TR-related genes (*GCM1*, *CGA*, *CGB*, *HSD17B1*, *CYP11A1*, *PGF*, *ESRRB*, *GATA2*, *GATA3*, *KRT19*) in TROP2^+^ cells (black bars), TROP2.^−^ cells (white bars), human ESC (ESC, green bars), and JAR cell line (JAR, blue bars). Gene expression values are reported with the highest expression set to 1 and all others relative to this (**G**)

After 2 days of co-culture in PTFE micro-bioreactors, chemically induced TR-like cells and ICM-like spheroids self-assembled in single 3D spherical structures (Fig. 4A). At the end of 7-day culture period, epiBlastoids displayed uniform round shape with sizes ranging from 100 to 200 µm, regardless of the sex of the fibroblasts. More in detail, morphometric analysis indicated that 78.67% of spheroids exhibited a diameter of 150–200 μm, while 21.33% of 100–150 μm (Fig. 4B).

Immunostaining studies showed CDX2^+^ cells localized in out layer of the epiBlastoids, while OCT4 was expressed by cells of the inner compartment (Fig. 4C). In addition, immunolocalization and western blot analyses demonstrated that TROP2^+^ cells were characterized by YAP nuclear accumulation, while TROP2^−^ cells by cytoplasmic compartmentalization of the molecule (Fig. 4D–F).

These morphological observations were also supported by molecular analyses indicating that TROP2^+^ cells expressed the TR markers *GCM1*, *CGA*, *CGB*, *HSD17B1*, *CYP11A1*, *PGF*, *ESRRB*, *GATA2*, *GATA3*, and *KRT19*, while TROP2^−^ cells transcribed for the pluripotency-related genes *OCT4*, *NANOG*, *REX1*, *SOX2*, *KLF17*, *PRDM14*, and *DPPA2* (Fig. 4G).

## Discussion

The possibility to successfully recreate in vitro blastocyst-like structures using embryonic stem cells (ESCs) or induced pluripotent stem cells (iPSCs) has been recently demonstrated both in the mouse [29–32] and the human [7–12]. In the present manuscript, we describe a novel method that combines, for the first time, the use of chemical stimuli and mechanical cues to generate in vitro 3D spherical structures phenotypically similar to natural embryos.

More in detail, in the first step, we induce a high permissivity window in adult dermal fibroblasts isolated from human healthy individuals, using 5-aza-CR which has been previously demonstrated to reactivate pluripotency-related genes [33–36], to induce a global DNA hypomethylation [16–24, 37–46] and to modulate ten-eleven translocation (TET) gene transcription [23]. In agreement with this, in the present work, adult dermal fibroblasts exposed to 5-aza-CR for 18 h lost their typical elongated morphology and acquired a round or oval shape, with larger nuclei and granular, vacuolated cytoplasm. These morphological changes closely resemble those previously identified in ESCs [47, 48] and iPSCs [49], suggesting the acquisition of features distinctive of a high plasticity phenotype. This was accompanied also by the onset of the main pluripotency-related gene transcription, namely, *OCT4*, *NANOG*, *REX1*, *SOX2*, *KLF17*, *PRDM14*, and *DPPA2*, which were originally undetectable in untreated fibroblasts (untreated fibroblasts), and by a significant down-regulation of the fibroblast-related markers *VIM* and *THY1*, further supporting previously published reports that described 5-aza-CR ability to induce a pluripotent-like phenotype hypomethylation [16–24, 37–46]. Taking advantage of the acquired high permissivity window, cells were readdressed towards the TR lineage, using an induction cocktail containing BMP4 in combination with activin/nodal and FGF2 signaling inhibitors. This differentiation medium has been previously shown to drive cells towards the TR phenotype in both human [27, 50–58] and pig [14, 28], with the acquisition of a tight adherent epithelial morphology, round shape, and nuclei, as well as well-defined borders. Consistent with this, immunocytochemical results indicated a high conversion efficiency (~ 80%), which is similar to that scored in human reprogrammed iPSCs differentiated towards TR lineage [27, 56, 59, 60] as well as in TR-like cells obtained from epigenetically converted porcine adult dermal fibroblasts [28]. In addition, the obtained TR-like cells showed active transcription for the TR mature markers *GCM1*, *CGA*, *CGB*, *HSD17B1*, *CYP11A1, PGF*, *ESRRB*, *GATA2*, *GATA3*, and *KRT19*, indicating the activation of the main molecular pathways distinctive of the newly acquired phenotype.

In the second step, we combined epigenetic and mechanosensing-related stimuli to generate ICM-like spheroids. To this purpose, erased adult dermal fibroblasts were encapsulated into PTFE micro-bioreactors to promote 3D cell rearrangement and boost pluripotency. Beside the morphological changes induced by exposure to 5-aza-CR, previously shown to be related to high plasticity, the use of the PTFE micro-bioreactors allowed cells to self-assemble and form multicellular spheroids, displaying a uniform size geometry. This well fits with previous observations indicating that the use of micro-bioreactors efficiently encourages 3D cell aggregation [61, 62], which, in turn, has the ability to support the induction of a pluripotent state and to boost its maintenance [16]. In agreement with this and consistently with our previous study [23], 86.31 ± 4.13% of cells were immune-positive for OCT4 and the generated ICM-like spheroids actively and steadily transcribed for the pluripotency-related genes *OCT4*, *NANOG*, *REX1*, *SOX2, KLF17*, *PRDM14*, and *DPPA2*, and down-regulated *VIM* and *THY1* genes.

In the third and last step of the protocol, we co-cultured TR-like cells with ICM-like spheroids in the PTFE micro-bioreactor for 2 days, to favor the formation of a single 3D spherical structure, composed by the two cell components. We subsequently transferred the newly generated aggregates into microwells and cultured them in a commercially available medium for further 5 days, to encourage epiBlastoid formation. Encapsulation of two different cell types has been previously reported by Jara et al. who applied this approach for the production of pancreatic islet-like structures in vitro [63]. These authors described encapsulation ability to stabilize 3D cell aggregation, maintain differentiation, and support functional activities. In the present manuscript, upon these specific culture conditions, TR-like cells and ICM-like spheroids were able to organize into structures with uniform round shape displaying a diameter ranging from 100 to 200 µm. In particular, morphometric analysis demonstrated that 78.67% of the spheroids exhibited a diameter ranging from 150 to 200 μm, with only 21.33% from 100 to150 μm. These results well fit with the average reported values of natural blastocysts that measure 175–211 μm and with the parameters and criteria reported in Table 2 and currently used to define human blastoid models. In addition, immunostaining studies demonstrated CDX2^+^ cells externally localized, to surround the epiblastoid, and OCT4^+^ cells closely assembled within them, showing that our protocol induced cells to spontaneously organize into spheroid complexes displaying TR-like cells homogenously distributed in the outer layer of the structures and ICM-like aggregates confined to the internal compartment. Consistent with this, sorting of epiBlastoid-derived cell suspensions, using the surface TR marker TROP2, led to the obtainment of two distinct cell populations: one consisting of TROP2^+^ cells and another containing TROP2^−^ cells. The first cell type actively transcribed for *GCM1*, *CGA*, *CGB*, *HSD17B1*, *CYP11A1*, *PGF*, *ESRRB*, *GATA2*, *GATA3*, and *KRT19*, genes, indicating that TROP2^+^ cells maintained a transcription pattern typical of TR cells. In addition, TROP2^+^ cells displayed a distinct compartmentalization of YAP which was mainly accumulated in the nucleus. In agreement with this, several studies reported the direct involvement of the Hippo pathway in the blastocyst formation, highlighting the key role played by the TEAD4/WWTR1/YAP1 complex in promoting *CDX2* expression during outside cell maturation to TR [64–78]. On the other hand, and in line with this, TROP2^−^ cells showed cytoplasmic retention of YAP and expressed the pluripotency-related genes *OCT4*, *NANOG*, *REX1*, *SOX2*, *KLF17*, *PRDM14*, and *DPPA2*.

**Table 2:** Summary of the parameters and criteria to define blastocyst-like in vitro model

Starting cell population	Induction molecules	Time(days)	Diameter (μm)	Cell lineages	Reference
Human blastocyst	n.a	5–7 days	175–211	EPI-, TE-, and PE-like cells	n.a
Embryonic stem cells	Trophoblast: PD0325901, A83-01, SB590885, WH-4–023, IM-12, CHIR99021, SB431542, recombinant human LIF, EGF, L-ascorbic acid, and VPA Hypoblast: bFGF, Activin A, and CHIR99021	6.5–11.5 days	50–319	EPI-, TE-, and HYPO-like cells	Leqian Yu et al. 2021
	PD0325901, A83-01	3–4 days	100–400	Not reported	Ayaka Yanagida et al. 2021
	CHIR99021, Y27632, BMP4, FGF2, and A83-01	5–6 days	Not reported	Not reported	Berna Sozen et al. 2021
	PD0325901, A83-01, LPA, Lif, Y-27632	3–4 days	150–250	TE-, EPI-, and PE- like cells	Harunobu Kagawa et al. 2022
Induced pluripotent stem cells	CHIR99021, A83-01, SB431542, valproic acid, EGF, BMP4	6 days	107–216	EPI-, TE-, and PE-like cells	Xiaodong Liu et al. 2021
	PD0325901, A83-01	3–4 days	100–400	Not reported	Ayaka Yanagida et al. 2021
	Trophoblast: BMP4, Y-27632 PSCs: LIF, CHIR99021, (S)-( +)-Dimethindene maleate, minocycline hydrochloride, IWR endo-1 and Y-27632 IVC: β-estradiol, progesterone	5–6 days	150–260	TE-, EPI-, and PE- like cells	Yong Fan et al. 2021
	PD0325901, A83-01, LPA, LIF, Y-27632	3–4 days	150–250	TE-, EPI-, and PE- like cells	Harunobu Kagawa et al. 2022
Epigenetically erased cells	Trophoblast: BMP4, A83-01, PD173074 PSCs: LIF, bFGF IVC: PODS Activin A	7 days	100–200	TE-, EPI—like cells	This work

Altogether, the procedure here described allows for an efficient in vitro generation of human epiBlastoids, starting from easily accessible adult dermal fibroblasts and avoiding the use of techniques that require retroviral gene transfection. Considering that the access to human embryo is subjected to tight regulation around the globe due to ethical concerns, the production of epiBlastoid models may overcome some and could find useful application in the assisted reproduction field, for the identification of the most adequate culture conditions and/or for peri- and early post-implantation investigations. In addition, the use of 3D micro-bioreactors may also represent a notable breakthrough in culture system technologies applied to reproduction and may constitute an advantageous micro-environment for long-term culture of blastoids, gastruloids, or organoids.

## Data Availability

The data presented in this study are available on request from the corresponding author.
