# Correction to: Combination of epigenetic erasing and mechanical cues to generate human epiBlastoids from adult dermal fibroblasts

**DOI:** 10.1007/s10815-023-02928-3

**Published:** 2023-09-12

**Authors:** Georgia Pennarossa, Sharon Arcuri, Teresina De Iorio, Sergio Ledda, Fulvio Gandolfi, Tiziana A. L. Brevini

**Affiliations:** 1 Department of Veterinary Medicine and Animal Science, Center for Stem Cell Research, Laboratory of Biomedical Embryology and Tissue Engineering, Università Degli Studi Di Milano, 26900 Lodi, Italy; 2 Department of Veterinary Medicine, University of Sassari, 07100 Sassari, Italy; 3 Department of Agricultural and Environmental Sciences ‑ Production, Landscape, Agroenergy, Università degli Studi di Milano, 20133 Milan, Italy

**Correction to: Journal of Assisted Reproduction and Genetics 40(5): 1015-1027**

https://doi.org/10.1007/s10815-023-02773-4.

Fig. 4 in the original version of this article has been replaced.

The original article has been corrected.

